# Anesthetic Challenges in a Pregnancy Case With Congenital Kyphoscoliosis

**DOI:** 10.7759/cureus.61269

**Published:** 2024-05-28

**Authors:** Shilpa Deshmukh, Dipanjali Mahanta, Yukti Roshni, Subhashree Jena

**Affiliations:** 1 Anaesthesiology, Dr. D. Y. Patil Medical College, Hospital and Research Centre, Dr. D. Y. Patil Vidyapeeth, Pune (Deemed to be University), Pune, IND; 2 Anaesthesiology, GNRC (Guwahati Neurological Research Centre) Hospitals, Guwahati, IND

**Keywords:** cesarean section, spinal anaesthesia, pregnancy, congenital spinal deformity, kyphoscoliosis

## Abstract

Kyphoscoliosis is a well-known spinal deformity. The abnormal curvature in both the coronal and sagittal planes presents unique challenges during pregnancy. This case discusses the management of a 27-year-old primigravida with thoracolumbar kyphoscoliosis, who underwent an emergency cesarean section at 39.3 weeks of gestation. An interdisciplinary team consisting of an obstetrician, pulmonologist, orthopedic surgeon, anesthesiologist, and physiotherapist collaborated in her care. In such cases, successful outcomes require a tailored approach that prioritizes maternal-fetal well-being and minimizes potential complications associated with complex spinal deformity during pregnancy and childbirth.

## Introduction

Kyphoscoliosis is a spinal deformity characterized by an abnormal curvature of the spine in both the coronal and sagittal planes due to disruption of the balance between structural and dynamic components or neuromuscular elements of the spine and symmetry of the body as a whole [1]. Kyphoscoliosis can have significant implications for women during pregnancy as the spinal deformity can pose unique challenges and potential complications. Pregnancy causes considerable strain on the musculoskeletal system due to the increasing weight of the growing fetus and the hormonal changes that occur during pregnancy [2]. For women with kyphoscoliosis, the existing spinal deformity adds an additional layer of complexity, which leads to increased back pain, respiratory difficulties, and potential complications during labor and delivery [3].

One of the primary concerns in kyphoscoliosis and pregnancy is the impact on cardio-respiratory function. The spinal curvature can limit the expansion of the thoracic cavity, which can cause respiratory compromise, particularly as the growing fetus exerts additional pressure on the diaphragm, leading to shortness of breath, decreased oxygen saturation, and an increased risk of respiratory complications during pregnancy and labor [3]. The cardiac output increases to about 40% by the end of the first trimester and 50% above nonpregnant levels by the third trimester, which is achieved by an increase in both heart rate and stroke volume. 

The abnormal spinal curvature can affect the positioning of the fetus within the uterus, potentially leading to complications such as fetal malposition or difficulty during the descent of the fetus through the birth canal [4]. This increases the incidence of cesarean section to ensure the safety of both the mother and the baby.

The additional strain on the spinal column and surrounding muscles can exacerbate back pain and discomfort, making it essential for appropriate pain management and physiotherapy to alleviate the symptoms [5,6].

Multidisciplinary care, involving obstetricians, anesthesiologists, orthopedic specialists, and physiotherapists, is considered for women with kyphoscoliosis during pregnancy. Careful monitoring, prenatal planning, and appropriate interventions are essential to ensure the best possible outcomes for both the mother and the developing fetus.

## Case presentation

**Figure 1:**
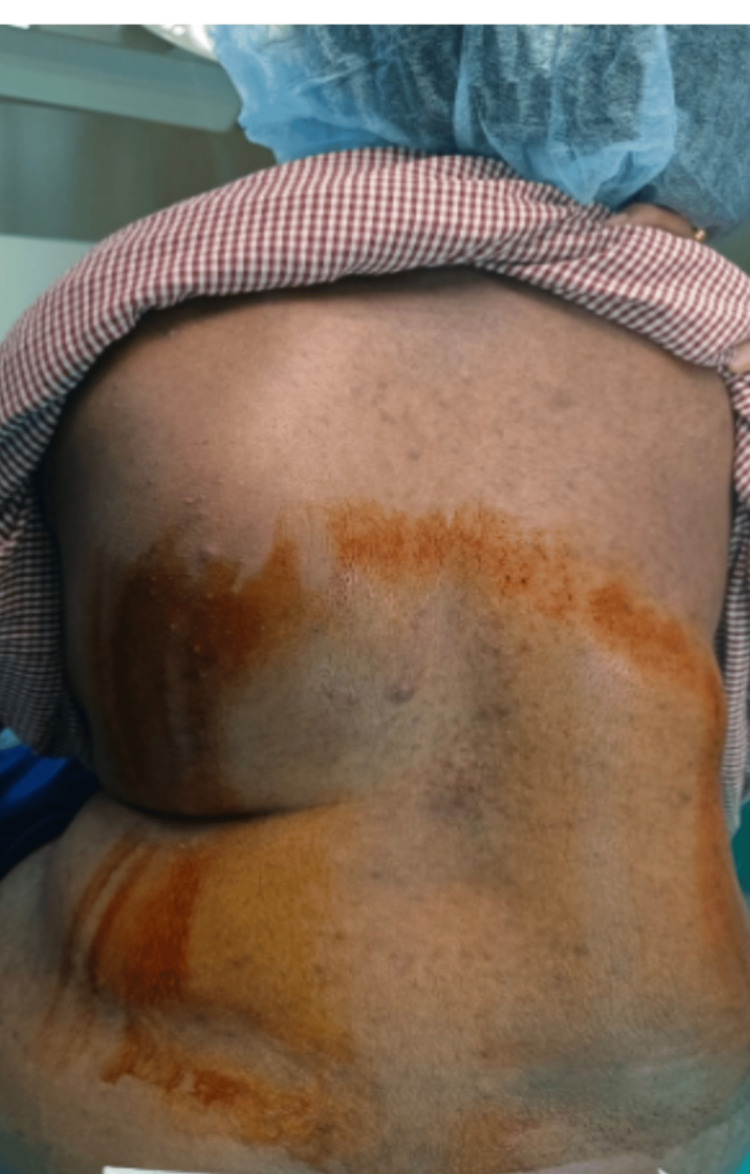
Kyphoscoliotic patient’s spine prepared for giving spinal anesthesia in a sitting position

A 27-year-old, diminutive primigravida, with significant “thoraco-lumbar” kyphoscoliosis weighing 70 kgs, height 140 cms, and BMI 35.7, presented to our tertiary care center at 39.3 weeks of gestation in labor. The patient was vitally stable and afebrile on physical examination. On auscultation, heart sounds were normal, with decreased bilateral air entry on the infra-mammary and infra-scapular regions with no adventitious sounds. The airway examination revealed Mallampati criteria 2 with a reduced thyromental distance and a short neck with an adequate range of movements. The examination of the spine revealed a congenital spinal deformity at the thoracolumbar region (Figure 1).

All routine laboratory investigations, including ECG and thyroid function tests, were normal, except for hemoglobin, which was 9.5 g/dL. 

**Figure 2:**
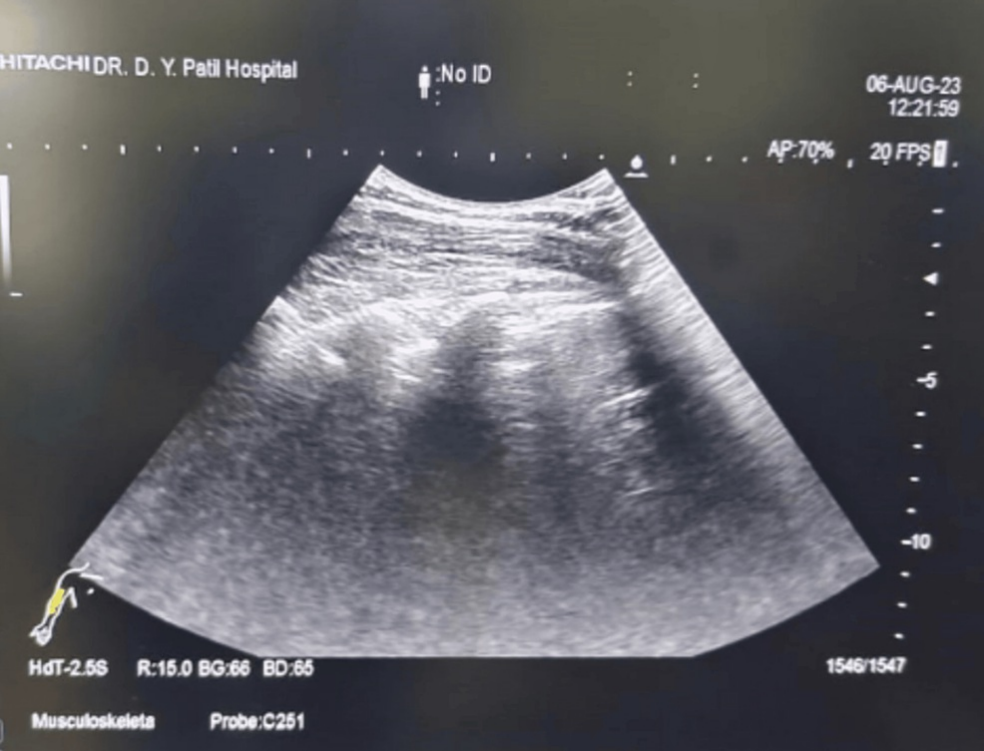
Ultrasonographic image of patient's spine in coronal and sagittal plane using a curved array probe

The patient was taken for an emergency cesarean section in view of active labor and maternal exhaustion. The patient was given aspiration prophylaxis and antibiotics prior to induction. An ultrasound scan (using a curved array probe) of the entire spine was performed in the sitting position, examining both longitudinal and transverse planes at different intervertebral levels (Figure 2).

**Figure 3:**
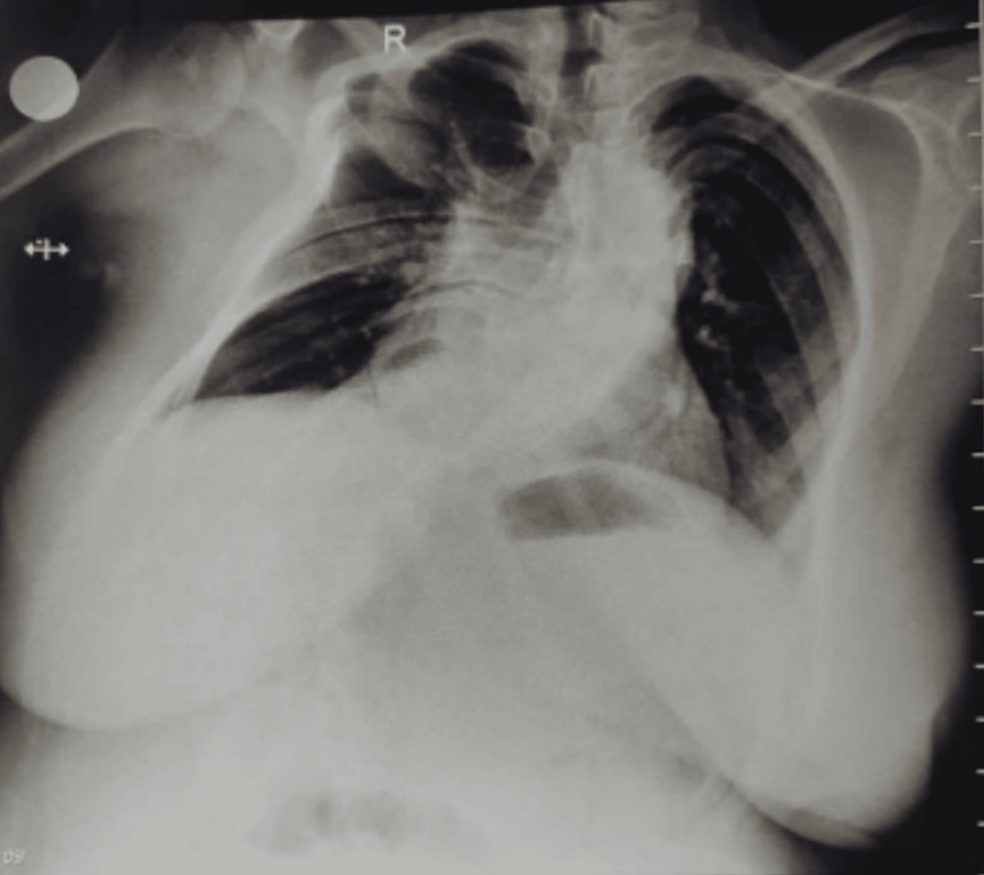
Postoperative posteroanterior chest radiographic view of kyphoscoliotic patient

Scanning in the longitudinal parasagittal plane revealed the spine to be curved to the left at the thoracolumbar level. In the parasagittal oblique view, the spinous process of L3 was identified. Then the probe was rotated 90 degrees and moved in cephalad direction to visualize L2-L3 intervertebral space with its structures. Once the best possible image was captured, the transducer tip was stabilized, and a horizontal line across the points joining the midpoints of the right and left lateral surfaces of the probe was drawn. A vertical line was drawn from the midline of the probe. The puncture site was marked at the intersection of these two lines. Under all aseptic precautions, a 23-G spinal needle was inserted at the level of L2-L3 intervertebral space. After two redirections, the subarachnoid space was confirmed with adequate and free flow of cerebrospinal fluid (CSF). A subarachnoid block was given with 2.0 mL of 0.5% bupivacaine heavy, and the patient was made to lie down (Figures 1-2). An adequate motor blockade was achieved within three minutes of induction. The sensory blockade on the right side was achieved at T6 within five minutes of induction, whereas on the left side took seven minutes for the level to reach T6. The patient was hemodynamically stable throughout the surgery. A male child weighing 2.8 kilograms was delivered and shifted to the mother’s side after confirming an adequate APGAR (appearance, pulse, grimace, activity, and respiration) score. Postoperatively, the BP was recorded as 130/80 mmHg, with a pulse rate of 80/min, respiratory rate of 20/min, and saturation of 99% on room air without any significant postpartum bleeding. Postoperatively, the patient was shifted to the intensive care unit for close monitoring of respiratory complaints for a period of 24 hours. The patient regained motor power and sensations in both the lower limbs within six hours of the procedure. The opinions of a pulmonologist and an orthopedic surgeon were obtained in view of symptomatic kyphoscoliosis. The pulmonologist advised a chest X-ray (PA view), pulmonary function test (PFT), incentive spirometry, and chest physiotherapy (Figure 3).

The patient was unable to perform PFT. The orthopedic surgeon advised conservative management with adequate physiotherapy. On postoperative day 2, the patient was shifted to the ward. The physiotherapist gave chest physiotherapy and assisted the patient with mobilization. The patient was discharged on postoperative day 7 in stable condition, with instructions for continued physiotherapy and follow-up.

## Discussion

The patient's spinal deformity and thoracic cage abnormalities predisposed her to respiratory compromise and increased risk of complications during labor and delivery. Pregnancy may exacerbate the severity of spinal curvature and cardiorespiratory abnormalities in patients with uncorrected scoliosis. The abnormal spinal curvature and chest wall deformity can lead to restrictive lung disease, decreased functional residual capacity, and ventilation-perfusion mismatch, which can worsen as the pregnancy progresses and the growing fetus exerts additional pressure on the diaphragm. In this case, the patient's dyspnea on exertion and decreased air entry on examination indicated the respiratory challenges associated with her deformity. Additionally, abnormal spinal curvature can affect the positioning and descent of the fetus during labor, increasing the risk of fetal malposition, obstructed labor, and fetal distress. These factors likely contributed to the prolonged labor course and the eventual need for an emergency cesarean section in this case. Kyphoscoliotic patients who present with increased peripheral resistance (PVR) may not be able to achieve increased outputs without further increments in vascular pressures, placing an intolerable load on the right ventricle and precipitating heart failure. Fixed pulmonary hypertension, unresponsive to supplemental oxygen therapy, carries a grave maternal prognosis and is an indication for termination of pregnancy. Maternal death at the time of delivery or immediate postpartum is common in parturients with pulmonary hypertension [7,8].

In kyphoscoliotic patients, the action of regional anesthesia may be patchy or fail, due to anatomical changes in the spine, lack of proper placement of local anesthetic, drug incompatibility, drug density, and drug defects [9]. The next option available is general anesthesia. The decision to proceed with an emergency cesarean section under regional anesthesia was a judicious choice, as general anesthesia could have exacerbated the patient's respiratory compromise and posed additional risks [10]. Postoperatively, close monitoring, respiratory support, and physiotherapy were crucial in facilitating a smooth recovery and preventing complications such as atelectasis, pneumonia, and thromboembolic events [11]. Such patients should ideally be planned for as elective cases, where thorough preoperative evaluation and optimization can be ensured.

## Conclusions

To conclude, kyphoscoliosis poses unique challenges during pregnancy and childbirth. The case highlights the successful use of spinal anesthesia in a patient with kyphoscoliosis for an emergency cesarean section. The anesthetic management must consider the well-being of both the mother and the fetus, as the need for anesthesia in pregnant women with scoliosis is more critical than in the normal parturient. The case also emphasizes the importance of thorough preoperative evaluation, including airway assessment, examination of the spine, and the need for adequate preparation to prevent potential complications.
